# The effect of atosiban on pregnancy outcomes in different FET cycles: a single-center matched retrospective cohort study

**DOI:** 10.3389/fendo.2025.1547694

**Published:** 2025-06-24

**Authors:** Yu Yang, Rong Luo, Shilei Qin, Wenkai Zhu, Minyan Yang, Dandan Zhu, Ping Zhang, Jia Wang, Hongshan Ge

**Affiliations:** ^1^ Department of Reproductive Medicine, The Affiliated Taizhou People’s Hospital to Nanjing Medical University, Taizhou, China; ^2^ Second Affiliated Hospital, School of Medicine, Zhejiang University, Hangzhou, China; ^3^ The Affiliated Taizhou People’s Hospital to Nanjing Medical University, Taizhou, China

**Keywords:** atosiban, live birth rate, frozen-thawed embryo transfer, assisted reproduction, endometrial peristalsis

## Abstract

**Background:**

The debate over the clinical role of atosiban in assisted reproduction continues. The purpose of our study was to explore the efficacy of atosiban on pregnancy outcomes of patients undergoing frozen embryo transfer.

**Methods:**

A total of 1615 frozen embryo transfer cycles between 1 January 2019 and 31 December 2022 were included in this retrospective cohort study. Patients were divided into two groups based on the administration of atosiban before frozen-thawed embryo transfer (FET): the atosiban group (n=339) and the control group (n=1276). The primary outcome was live birth, while the secondary outcomes were biochemical pregnancy, clinical pregnancy, abortion, and ectopic pregnancy.

**Results:**

After propensity score matching (PSM), both univariable and multivariable analyses showed atosiban was not linked to an increased likelihood of biochemical pregnancy or clinical pregnancy, nor a reduced risk of abortion or ectopic pregnancy (p>0.05). When controlling for confounding factors, maternal age (OR, 0.95; 95% CI, 0.91-0.98; p=0.004), history of failed ETs (1: OR, 0.72; 95% CI, 0.53-0.99; p=0.040; ≥2: OR, 0.65; 95% CI, 0.46-0.92; p=0.015), embryo stage (OR, 2.45; 95% CI, 1.85-3.25; p=0.000) and endometrial thickness (OR, 1.12; 95% CI, 1.01-1.24; p=0.025) were found to be associated with the likelihood of live birth. No beneficial effect of atosiban was observed in any of the subgroups based on maternal age, number of previous embryo transfers (ETs), endometrial thickness, or embryo stage in the subgroup analysis of the primary outcome.

**Conclusion:**

These results suggested that adding atosiban in the standard FET cycles might not improve the live birth rate. To confirm this conclusion, more thorough, prospective randomized controlled studies of sizable sample sizes with good design are required.

## 1 Introduction

Over four decades have elapsed since the birth of the first *in vitro* fertilization (IVF) baby in the 1970s. Despite advanced progress in assisted reproduction technology (ART) over the past 40 years, embryo implantation remains the bottleneck of assisted reproduction. Embryo transfer (ET) is the final step of IVF-ET, the successful rate was just 23% per embryo transferred (1). There may be many possible factors, such as embryo quality, endometrial receptivity, etc. (2). Embryo quality accounts for around one-third of implantation failure while Suboptimal uterine receptivity accounts for two-thirds (3). A previous study (4) reported that the synchronization of embryo and endometrium is influenced by a variety of factors, such as embryonic and parental inheritance, anatomical factors, maternal immune system, endocrine environment, hematologic factors, and reproductive tract microorganisms. In recent years, a vast number of methods aimed at improving the success rate of IVF-ET through optimizing embryo transfer have been proposed, such as intrauterine infusion with hCG (5–9), hysteroscopy screening (10, 11), and endometrial implantation window detection done before ET (12).

In addition, rhythmic uterine contractions of the non-pregnant uterus have an important role in human reproduction (13). Contractions from the fundus to cervix are observed primarily in the early to mid-follicular phase and decrease as ovulation approaches. A switch in the direction of contractions occurs in the late follicular phase when waves from the cervix to fundus are observed (14–16). These progressive increased cervix-to-fundus contractions during the periovulatory period are thought to assist sperm ascension towards the distal end of the fallopian tubes, where fertilization takes place (14). In this phase, the frequency of uterine contractions is highest (17) and their direction is predominantly ipsilateral to the dominant follicle (18). After ovulation, opposing uterine contractions – defined as simultaneous contractions originating in the cervix and fundal area – appear. The opposing uterine contractions serve to prevent the embryo from being expelled from the cervix or the tubes, providing nutrients, and positioning the embryo before implantation (15, 17).In the luteal phase, the uterus is in a quiescent state, providing an optimal environment for the implantation of the embryo (19, 20). Briefly, the function of uterine contractions seems to be two-fold: providing strong enough contractions to guide spermatozoa to the ovum during ovulation; and creating an optimal, quiescent environment for implantation of an embryo during the luteal phase. Failure in one of these functions can interfere with fertility (21). Some evidence shows that there is a negative association between contractions at the time of ET and the clinical outcome (19), thus, methods or medications that can reduce uterine contractions during ET are attractive options to improve IVF success rates.

Oxytocin receptors (OTRs) are abundantly expressed in the pregnant uterus (particularly during the second and third trimesters), while detectable expression is also present in non-pregnant uterine tissue at lower levels sufficient to mediate uterine contractions (22). Oxytocin receptor antagonists (including atosiban, barusiban, nolasiban, epelsiban, and retosiban) can compete with oxytocin (OT) of oxytocin receptors (OTRs) in uterine smooth muscle cells, decidual cells, and fetal membrane and inhibit OT-induced PGF2a and uterine activity (23). Atosiban is the best-known oxytocin antagonist commonly used to delay premature labor. Studies have revealed a six−fold increase in uterine contractility before ET in IVF cycles compared with that before ovulation in natural cycles (24). Furthermore, there is a considerable reduction in the frequency of uterine contractions from 16/4 minutes to 6–2.6/4 minutes in women undergoing ET following the administration of atosiban (25). However, whether the application of atosiban around ET can improve the clinical outcomes of IVF-ET remains controversial.

While some studies showed that atosiban could improve clinical outcomes after ET (25, 26), other research revealed that atosiban only benefited subjects with a history of implantation failure, especially recurrent implantation failure (RIF) (27–31). Furthermore, some researchers even demonstrated that the application of atosiban could improve neither the implantation nor clinical pregnancy rates of patients undergoing IVF treatment (32, 33).

Considering the inconsistent clinical role of atosiban for infertility people, this retrospective cohort study was conducted to discuss the effects of atosiban in the FET cycle. The results would provide a reliable basis to guide clinicians in the selection of medications during FET cycles.

## 2 Materials and methods

### 2.1 Study design and patients

This retrospective cohort study was reviewed and approved by the institutional review board of the Affiliated Taizhou People’s Hospital to Nanjing Medical University (KY 2024-166-01), conducted at the Department of Reproductive Medicine of the hospital from January 2019 to December 2022.

This study included patients who underwent IVF/ICSI cycles and had at least one frozen embryo transfer (FET) cycle. The exclusion criteria were as follows: (a) patients aged 40 years or older; (b) patients with chromosomal abnormalities; (c) pre-implantation genetic testing (PGT) cycles; (d) egg donation cycles; (e) untreated hydrosalpinx, uterine malformation, precancerous lesions, and malignant neoplasm; (f) cycles lost to follow-up; (g) cycles with critical data missing. The information on demographic characteristics, associated laboratory measurements, ET protocol, and pregnancy outcomes for each FET cycle was recorded from electronic medical records, including maternal age, infertility duration, gravidity, parity, history of failed ETs, basic serum hormone levels, body mass index (BMI), anti-mullerian hormone (AMH), FET treatment procedures and reproductive outcomes. Finally, a total of 1,615 FET cycles were categorized into two groups based on the administration of atosiban before FET: the atosiban group (n=339) and the control group (n=1276). The decision to use atosiban was made based on a shared decision-making process involving the patient and the physician. Patients were informed about the potential effects, side effects, and costs of atosiban, and the final decision was influenced by the patient’s preferences, financial situation, and the physician’s clinical judgment and prescribing practices. The patients in the treatment group received intravenous atosiban 30 minutes before FET with a bolus dose of 6.75 mg (Tractocile, Ferring Pharmaceuticals, Kiel, Germany), within 1–2 min infusion time.

### 2.2 Frozen-thawed embryo transfer

The endometrium was prepared for the subsequent FET cycles, using the natural or artificial protocol, as previously described (34)[1]. Briefly, for ovulatory patients, FET was performed in the natural cycle. Patients underwent transvaginal ultrasound monitoring on days 2–5 of their menstrual cycle. Ovulation was triggered with human chorionic gonadotropin (hCG) (Chorionic Gonadotropin For Injection; Livzon (GROUP) Pharmaceutical Co., Ltd.) when the dominant follicle reached 18 mm. FET was performed 4 to 7 days after the hCG injection, depending on the embryo stage. Luteal phase support was provided using 40 mg of intramuscular progesterone (Progesterone Injection; Tianjin Kingyork Pharmaceuticals Co.) and 40 mg of oral dydrogesterone (Duphaston; Abbott Biologicals B.V.). For anovulatory women, FET was done based on programmed protocol. Whether downregulation was administered prior to hormone replacement (HR) was based on shared decision-making, considering patient preferences, financial status, and the physician’s clinical judgment. HR cycles were conducted with increasing doses of oestradiol valerate (Progynova; Bayer AG), administered at 2 to 8 mg daily for a minimum of 12 days. If endometrial thickness reached 7mm or more and there were no dominant follicles, both estradiol and luteal phase support were given. FET was performed after 3 to 6 days of progesterone supplementation. No more than 2 embryos were transferred per FET cycle. If pregnancy was confirmed, luteal support was continued until the 10th to 12th week of pregnancy.

### 2.3 Outcome measures

The primary outcome was live birth and the secondary outcomes were biochemical pregnancy, clinical pregnancy, abortion, and ectopic pregnancy. Live birth was defined as the delivery of one or more living infants at 28 weeks’ gestation or later. Biochemical pregnancy was defined as a positive serum β-hCG result 14 days after FET. Clinical pregnancy was defined as the presence of one or more gestational sacs with visible cardiac activity on transvaginal ultrasound. Abortion was defined as the spontaneous loss of a clinically recognized pregnancy within 28 weeks of gestation. Ectopic pregnancy was defined as the implantation of the embryo outside the uterine cavity.

### 2.4 Statistical analysis

Data were expressed as the numbers (%) for dichotomous variables and median (interquartile range, IQR) for continuous variables as no continuous variables meet the normality criteria of distribution. The missing rates of several demographic characteristics and laboratory measurements were less than 3% and we imputed missing data using multiple imputation.

To address the differences in the baseline characteristics in the two groups, we utilized PSM to mitigate the variations in attributes and reduce the influence of potential confounding factors and selection bias. The variables considered in the PSM model included maternal age, infertility duration, gravidity, parity, number of ET failures, BMI, basal hormone levels, AMH, embryo developmental stage at cryopreservation, number of embryos transferred, and endometrial thickness. Patients in the atosiban group were paired with patients in the control group using the nearest-neighbor method with a caliper width of 0.2 without replacement, in a ratio of 1:2.

Before and after PSM, Mann–Whitney U test, chi-square test or Fisher’s exact test were conducted for comparison between groups. For adjustment purposes, variables in the matched cohort dataset that had a p<0.05 in the univariable analysis were added to the multivariable analysis. Crude and adjusted odds ratios (ORs) with 95% confidence intervals (CIs) are used to display effect estimates.

Subgroup analyses in the matched cohort were based on maternal age (<35 vs ≥35 yr), number of failed ETs (0 vs 1 vs ≥2), endometrial thickness (<8 vs ≥8 mm), and embryo stage (cleavage stage vs blastocyst). A significance level of p<0.05 was used to determine statistical significance. Statistical analyses were conducted using SPSS 27.0 (IBM Corp., USA) and R 4.3.0 (R Foundation, Vienna, Austria).

## 3 Results

### 3.1 Baseline characteristics

The included FET cycles were divided into the atosiban group and the control group based on whether atosiban was used, with 339 and 1276 cycles in each group, respectively. After PSM, 333 cycles with atosiban administration before FET were matched with 646 cycles without atosiban administration. Standardized mean differences (SMDs) for covariates were below the threshold of 0.1, indicating adequate balance between groups (**Supplementary Figure S1**). In the original unmatched cohort, there were differences in parity, number of failed ETs, endometrial thickness, and developmental stages at cryopreservation for transferred embryos between the two groups (p<0.05) (**Table 1**). In the PSM-matched cohort, no significant differences were observed between groups (p>0.05) (**Table 1**).

**Table 1:** Baseline characteristics before and after propensity score matching.

Characteristics	Before PSM			After PSM		
	Atosiban (n=339)	Control (n=1276)	p-value	Atosiban (n=333)	Control (n=646)	p-value
Age (yr)	30 (27, 33)	30 (27, 33)	0.245	30 (27, 33)	30 (27, 33)	0.731
<35	277 (81.7)	1028 (80.6)	0.634	271 (81.4)	541 (83.7)	0.351
≥35	62 (18.3)	248 (19.4)		62 (18.6)	105 (16.3)	
Duration of infertility (yr)	2.4 (1.5, 4)	2.5 (1.5, 4)	0.733	2.4 (1.5, 4.0)	2.5 (1.5, 4.0)	0.930
Primary infertility	185 (54.6)	645 (50.5)	0.188	180 (54.1)	348 (53.9)	0.956
Gravidity	0 (0, 1)	0 (0, 1)	0.211	0 (0, 1)	0 (0, 1)	0.971
Parity	0 (0, 0)	0 (0, 0)	0.017	0 (0, 0)	0 (0, 0)	0.504
No. of failed ETs	1 (0, 2)	0 (0, 1)	0.000	1 (0, 2)	1 (0, 1)	0.904
0	148 (43.7)	671 (52.6)	0.000	148 (44.4)	268 (41.5)	0.252
1	97 (28.6)	424 (33.2)		97 (29.1)	222 (34.4)	
≥2	94 (27.7)	181 (14.2)		88 (26.4)	156 (24.1)	
BMI (Kg/m2)	22.1 (20.4, 25.4)	22.7 (20.5, 25.7)	0.146	22.1 (20.4, 25.5)	22.6 (20.4, 25.4)	0.559
Basal FSH (IU/L)	6.60 (5.58, 7.86)	6.73 (5.50, 8.08)	0.512	6.65 (5.61, 7.88)	6.64 (5.44, 8.00)	0.613
Basal E_2_ (pmol/ml)	116.00 (89.87, 152.03)	116.83 (87.43, 151.48)	0.523	116.00 (89.52, 152.02)	119.68 (90.24, 154.13)	0.663
Basal LH (IU/L)	4.43 (2.96, 6.45)	4.52 (3.25, 6.38)	0.387	4.43 (2.97, 6.49)	4.28 (3.17, 6.16)	0.752
AMH (ng/ml)	3.89 (2.26, 6.37)	3.86 (2.12, 6.41)	0.636	3.89 (2.27, 6.43)	3.87 (2.12, 6.14)	0.392
Embryo type						
Cleavage stage	204 (60.2)	895 (70.1)	0.000	203 (61.0)	401 (62.1)	0.734
Blastocyst	135 (39.8)	381 (29.9)		130 (39.0)	245 (37.9)	
No. of embryos transferred						
1	44 (13.0)	215 (16.8)	0.084	43 (12.9)	75 (11.6)	0.553
2	295 (87.0)	1061 (83.2)		290 (87.1)	571 (88.4)	
Endometrial thickness (mm)	8.9 (8.4, 9.8)	9.2 (8.5, 10.1)	0.025	8.9 (8.5, 9.9)	9.0 (8.4, 9.8)	0.897
≥8mm	297 (87.6)	1138 (89.2)	0.413	294 (88.3)	564 (87.3)	0.658
<8mm	42 (12.4)	138 (10.8)		39 (11.7)	82 (12.7)	
Endometrial preparation protocol						
Programmed protocol	251 (74.0)	981 (76.9)	0.275	290 (87.1)	497 (88.1)	0.653
NC protocol	88 (26.0)	295 (23.1)		43 (12.9)	149 (11.9)	

PSM, propensity score matching; ET, embryo transfer; BMI, body mass index; FSH, follicle stimulating hormone; E2, estradiol; LH, luteinizing hormone; AMH, anti-mullerian hormone; NC, natural cycle.

### 3.2 Outcomes

Preliminary comparisons revealed no significant statistical differences in both primary and secondary outcomes between the two groups before and after PSM (p>0.05) (**Table 2**).

**Table 2:** Primary and secondary outcomes before and after propensity score matching.

Outcomes	Before PSM			After PSM		
	Atosiban (n=339)	Control (n=1276)	p-value	Atosiban (n=333)	Control (n=646)	p-value
Live birth	146 (43.1)	564 (44.2)	0.709	143 (42.9)	307 (47.5)	0.173
Biochemical pregnancy	194 (57.2)	746 (58.5)	0.682	189 (56.8)	402 (62.2)	0.097
Clinical pregnancy	182 (53.7)	690 (54.1)	0.899	178 (53.5)	376 (58.2)	0.155
Abortion	35 (19.2)	119 (17.2)	0.532	34 (19.1)	64 (17.0)	0.549
Ectopic pregnancy	1 (0.3)	7 (0.5)	0.558	1 (0.3)	5 (0.8)	0.368

PSM, propensity score matching.

In the PSM-matched cohort, atosiban use was not associated with a higher live birth rate in the univariable analysis (OR, 0.83; 95% CI, 0.64-1.08; p=0.170) as well as in the multivariable analysis (OR, 0.80; 95% CI, 0.61–1.06; p=0.124) (**Table 3**). After adjusting for confounding factors, maternal age (OR, 0.95; 95% CI, 0.91-0.98; p=0.004), history of failed ETs (1: OR, 0.72; 95% CI, 0.53-0.99; p=0.040; ≥2: OR, 0.65; 95% CI, 0.46-0.92; p=0.015), embryo stage (OR, 2.45; 95% CI, 1.85-3.25; p=0.000) and endometrial thickness (OR, 1.12; 95% CI, 1.01-1.24; p=0.025) were related to the probability of live birth (**Table 3**). For secondary outcomes, both univariable and multivariable analyses showed atosiban was not linked to an increased likelihood of biochemical pregnancy or clinical pregnancy, nor a reduced risk of abortion or ectopic pregnancy (p>0.05) (**Table 4**).

**Table 3:** Univariate and multivariate analysis of the association between atosiban and primary outcome (live birth) after propensity score matching.

Characteristics	Univariate analysis		Multivariate analysis	
	Crude OR (95%CI)	p-value	Adjusted OR (95%CI)	p-value
Atosiban	0.83 (0.64, 1.08)	0.170	0.80 (0.61, 1.06)	0.124
Age (yr)	0.92 (0.89, 0.95)	0.000	0.95 (0.91, 0.98)	0.004
Duration of infertility (yr)	0.92 (0.87, 0.98)	0.007	0.94 (0.88, 1.01)	0.075
Primary infertility	0.80 (0.62, 1.03)	0.082		
Gravidity	0.82 (0.74, 0.91)	0.000	0.89 (0.78, 1.02)	0.105
Parity	0.60 (0.44, 0.83)	0.002	0.89 (0.59, 1.37)	0.603
No. of failed ETs				
0	Reference		Reference	
1	0.62 (0.46, 0.83)	0.001	0.72 (0.53, 0.99)	0.040
≥2	0.47 (0.34, 0.65)	0.000	0.65 (0.46, 0.92)	0.015
Basal BMI (Kg/m2)	0.99 (0.96, 1.02)	0.549		
Basal FSH (IU/L)	0.98 (0.93, 1.03)	0.348		
Basal E2 (pmol/ml)	1.00 (1.00, 1.00)	0.978		
Basal LH (IU/L)	1.04 (0.99, 1.08)	0.095		
AMH (ng/ml)	1.05 (1.01, 1.09)	0.018	0.99 (0.95, 1.03)	0.619
Programmed protocol	1.05 (0.78, 1.41)	0.739		
Blastocyst transferred	2.66 (2.04, 3.47)	0.000	2.45 (1.85, 3.25)	0.000
Double embryo transferred	1.33 (0.90, 1.97)	0.156		
Endometrial thickness (mm)	1.14 (1.04, 1.25)	0.006	1.12 (1.01, 1.24)	0.025

OR, odds ratio; CI, confidence interval; ET, embryo transfer; BMI, body mass index; FSH, follicle stimulating hormone; E_2_, estradiol; LH, luteinizing hormone; AMH, anti-mullerian hormone.

**Table 4:** Univariate and multivariate analysis of the association between atosiban and secondary outcomes after propensity score matching.

Secondary outcomes	Univariate analysis		Multivariate analysis^a^	
	Crude OR (95%CI)	p-value	Adjusted OR (95%CI)	p-value
Biochemical pregnancy	0.80 (0.61, 1.05)	0.102	0.78 (0.59, 1.03)	0.077
Clinical pregnancy	0.82 (0.63, 1.07)	0.151	0.80 (0.61, 1.06)	0.120
Abortion	1.15 (0.73, 1.83)	0.547	1.22 (0.75, 1.98)	0.426
Ectopic pregnancy	0.40 (0.05, 3.44)	0.403	0.40 (0.05, 3.51)	0.406

a adjusted for age, duration of infertility, gravidity, parity, No. of failed ETs, embryo type, AMH, endometrial thickness; OR, odds ratio; CI, confidence interval.

In the subgroup analysis of primary outcome, no beneficial effect of atosiban was observed in the maternal age subgroups (<35 vs ≥35 yr), number of failed ETs subgroups (0 vs 1 vs ≥2), endometrial thickness subgroups (<8 vs ≥8 mm), and embryo stage subgroups (cleavage stage vs blastocyst) (p>0.05) (**Table 5**).

**Table 5:** Univariate and multivariate analysis of the association between atosiban and primary outcome (live birth) in the subgroups after propensity score matching.

Subgroups	Live birth	Crude OR (95%CI)	p-value	Adjusted OR (95%CI)	p-value
Age (yr)					
<35	403 (49.6)	0.83 (0.62, 1.11)	0.201	0.82 (0.60, 1.11)^a^	0.195
≥35	47 (28.1)	0.94 (0.47, 1.89)	0.859	0.64 (0.29, 1.44)^a^	0.283
No. of failed ETs					
0	226 (54.5)	0.86 (0.57, 1.29)	0.460	0.87 (0.57, 1.33)^b^	0.525
1	136 (42.6)	0.82 (0.50, 1.33)	0.413	0.78 (0.47, 1.31)^b^	0.348
≥2	88 (36.1)	0.75 (0.43, 1.30)	0.297	0.66 (0.37, 1.20)^b^	0.171
Embryo type					
Cleavage stage	222 (36.8)	0.86 (0.60, 1.22)	0.396	0.86 (0.60, 1.24)^c^	0.428
Blastocyst	228 (60.8)	0.74 (0.48, 1.15)	0.181	0.71 (0.45, 1.11)^c^	0.130
Endometrial thickness					
≥8mm	418 (48.8)	0.81 (0.61, 1.07)	0.136	0.81 (0.60, 1.09)^d^	0.158
<8mm	32 (26.4)	0.93 (0.39, 2.21)	0.869	0.71 (0.27, 1.92)^d^	0.503

a adjusted for duration of infertility, gravidity, parity, No. of failed ETs, embryo type, AMH, endometrial thickness;

b age, duration of infertility, gravidity, parity, embryo type, AMH, endometrial thickness;

c age, duration of infertility, gravidity, parity, No. of failed ETs, AMH, endometrial thickness;

d age, duration of infertility, gravidity, parity, No. of failed ETs, embryo type, AMH; OR, odds ratio; CI, confidence interval; ET, embryo transfer.

## 4 Discussion

The current study evaluated the effect of atosiban on pregnancy outcomes in the general population with different numbers of FET cycles. In this retrospective cohort study, our results did not reveal a positive effect of atosiban on pregnancy outcomes. The biochemical pregnancy, clinical pregnancy, and live birth rate were not improved in the atosiban group compared to the control group.

Uterine contraction is one of the basic elements of endometrial receptivity and plays an important role in the embryo implantation process. As the studies showed (35–37), a high level of estradiol (E2), in the ovarian stimulation cycle might stimulate oxytocin and prostaglandin (PG)F2a production from endometrial cells, which may produce strong and frequent uterine contractions. To avoid the effect of high estrogen, our study chose FET cycles rather than fresh cycles. A previous study (33) revealed no difference in the live birth rate between groups stratified by the frequency of endometrial peristalsis. On the other hand, measuring uterine contractions was time-consuming and had intra-variation between different observers, thus the accuracy of the measurement is questionable. So we did not measure endometrial peristalsis.

Patients who experienced constant transvaginal ultrasound supervision or rough manipulation may lead to a hyperactivated autocrine/paracrine OT/OTR system in the endometrial epithelium that can result in a high level of serum OT and PGF2a, thereby leading to a high uterine contraction (30). Thus, gentle manipulation during FET is one of the ways to reduce endometrial contractions. On the other hand, medications that reduce uterine contractions during ET are also attractive options to improve IVF success. Atosiban, the best-known combined oxytocin/vasopressin V1A antagonist, exerts its effect by competing with oxytocin to its receptors located in the myometrium, decidua, and fetal membranes. This competition diminishes the efficacy of oxytocin and reduces the intracellular calcium ion levels in myometrial cells, consequently inhibiting uterine contractions. In addition, it can boost endometrial perfusion (38, 39). In a preclinical study, atosiban had a good embryonic safety profile and did not affect the endocrine profile up to 50-fold therapeutic blood concentrations. It neither affected the survival of the 1‐cell rabbit embryo nor affected the hatched rabbit blastocysts percentage. It had no adverse influence on human spermatozoa during sperm motility bioassays (39). Another study shows that atosiban has no systemic toxicity, mutagenic effects, or carcinogenic effects (25).

Our results agreed with Buddhabunyakan et al. (40), who demonstrated that adding atosiban during FET did not reduce uterine peristalsis and improve pregnancy outcomes in the general population. One RCT compared the live birth rates in women receiving atosiban versus placebo in fresh ET cycles in which day 3 embryos were transferred. The results showed no significant improvement in pregnancy outcomes with atosiban as compared with placebo in the general population undergoing fresh ET cycles (32). In their opinion, atosiban can reduce the frequency and amplitude of uterine contractions, but the proportion of cycles with uterine contractions of >3/min varied widely from 6.2% to 65.0%, and there was no difference between groups stratified by the frequency of endometrial peristalsis. In our multivariate analysis, maternal age, history of failed embryo transfers, embryo stage, and endometrial thickness emerged as significant covariates associated with live birth rates. To evaluate whether these variables might potentially modify the magnitude or direction of the association between the primary exposure (atosiban administration) and clinical outcomes, while simultaneously assessing the robustness of our primary findings across distinct patient populations, we conducted subgroup analyses stratified by these key parameters. In these analyses, atosiban did not show statistically significant treatment benefits. He et al. (30) analyzed several subgroups of infertile women undergoing different numbers of ET cycles and found that the use of atosiban significantly increased clinical and implantation rates among IVF patients undergoing third or subsequent ET cycles, but no statistical significance was observed for the efficacy of atosiban in the first- and second-ET groups. They have found that low levels of serum oxytocin and PGF2α among patients undergoing the first or second ET cycles were associated with low uterine contractions, which implies a stable uterine environment and explains the lack of response to atosiban observed among these patients. Another longitudinal cohort study (29) with larger numbers of ET cycles was consistent with the results of He et al.’ study. However, our subgroup analysis revealed no difference in clinical outcomes between the two groups stratified by the number of FET cycles. Aneuploidy leads to the majority of preclinical pregnancy losses (41). As we know, they did not test the embryos’ aneuploidy status by PGT-A. As a result, patients with possible implantation of aneuploidy embryos were not excluded in those studies and this might have an impact on their results. This may explain why they got positive results. However, the recurrent implantation failure (RIF) subgroup in our study had a limited sample size, and given the retrospective nature of our study design, these findings should be interpreted as exploratory. Definitive confirmation through large-scale randomized controlled trials will be required to validate these observations.

Another reason why we did not observe any benefit of atosiban may be related to the regimen of atosiban infusion used in the present study, which was based on the study of He et al. (30). Atosiban is a very short-acting drug, so it was administered 30 min before the transfer with a bolus dose of 6.75 mg within 1–2 min infusion time in our center. Therefore, the reduction in uterine contractions may not last long enough after stopping the atosiban infusion to produce appreciable effects on the outcome measures. Since embryo implantation takes place 3 days after cleavage embryo transfer and 1 day after blastocyst transfer, a prolonged atosiban infusion over 1–2 days may be associated with a sustained reduction in uterine contractions after ET, leading to a higher live birth rate (33).

As expected, younger age, blastocyst transferring and a thicker endometrial improve the LBR after ET. Advanced maternal age may lead to a compromised competence of the oocytes/embryos because of defective physiological pathways, such as energy production and balance, metabolism, epigenetic regulation, cell cycle checkpoints, and increased meiotic missegregation (42, 43). The consequence of an aging egg is abnormal fertilization and development, such as polyspermy, division arrest, implantation failure, and miscarriage (44–46). Women with advanced maternal age would experience a poor ovarian response, fewer retrieved oocytes, lower fertilization rate, and embryo quality rates, reduced embryo implantation and pregnancy rates, and higher risks of miscarriage, preterm delivery rate, and birth defect rate (47, 48). Studies have shown that early cleavage embryos peristalsis in the uterine cavity and fallopian tube before implantation, leads to a higher rate of ectopic pregnancy. Moreover, they were more likely to induce uterine contractions than blastocysts (49–51). The culture process of blastocyst provides more morphogenetic information to identify and discard embryos with lower implantation potential, thus enabling self-selection of viable embryos. While blastocyst, appropriate age, and endometrial of moderate thickness also promote better embryo-endometrial synchrony (52–56).

However, several limitations of this study need to be acknowledged. Firstly, all participants were from one medical center. Therefore, selection bias could not be eliminated. Secondly, we did not track congenital abnormalities in newborns. Thirdly, this observational study included all patients who attended the study medical center during the study period, so no formal sample size calculations were conducted. Fourth, the sample size of some subgroups (such as older women or patients with recurrent implantation failure) was relatively limited, which may reduce statistical power and potentially underestimate or overestimate the true effect. Nevertheless, with the growing demand for fertility preservation among older patients, we anticipate that future studies will benefit from larger cohort sizes. Lastly, this was a retrospective study, and thus the associated limitations could not be avoided (e.g., selection bias, reporting bias, and incomplete or missing data). The original dataset lacked detailed documentation of specific infertility factors, compounded by the frequent co-existence of multiple infertility factors in our patient population. These limitations prevented meaningful subgroup analysis of individual infertility causes and their potential impact on treatment outcomes.

## 5 Conclusions

In summary, the use of atosiban given before FET did not improve the live birth rate in general patients. Similar results were found in patients stratified by maternal age, number of previous failed embryo transfers, embryo type, or endometrial thickness on embryo transfer day. The clinical value of using atosiban in conventional embryo transfer strategy needs to be further studied.

## Data Availability

The raw data supporting the conclusions of this article will be made available by the authors, without undue reservation.
