# Supplementary material for: The effect of atosiban on pregnancy outcomes in different FET cycles: a single-center matched retrospective cohort study
Source: Front Endocrinol (Lausanne). 2025 Jun 24;16:1547694. doi: 10.3389/fendo.2025.1547694 (PMC12234319; doi:10.3389/fendo.2025.1547694)

Supplementary Material

The effect of atosiban on pregnancy outcomes in different FET cycles: a single-center matched retrospective cohort study

*** Correspondence:** Hongshan Ge: hongshange@njmu.edu.cn and Jia Wang: 32735644@qq.com

Supplementary Figure 1. Standardized mean differences before and after matching between the atosiban and control groups

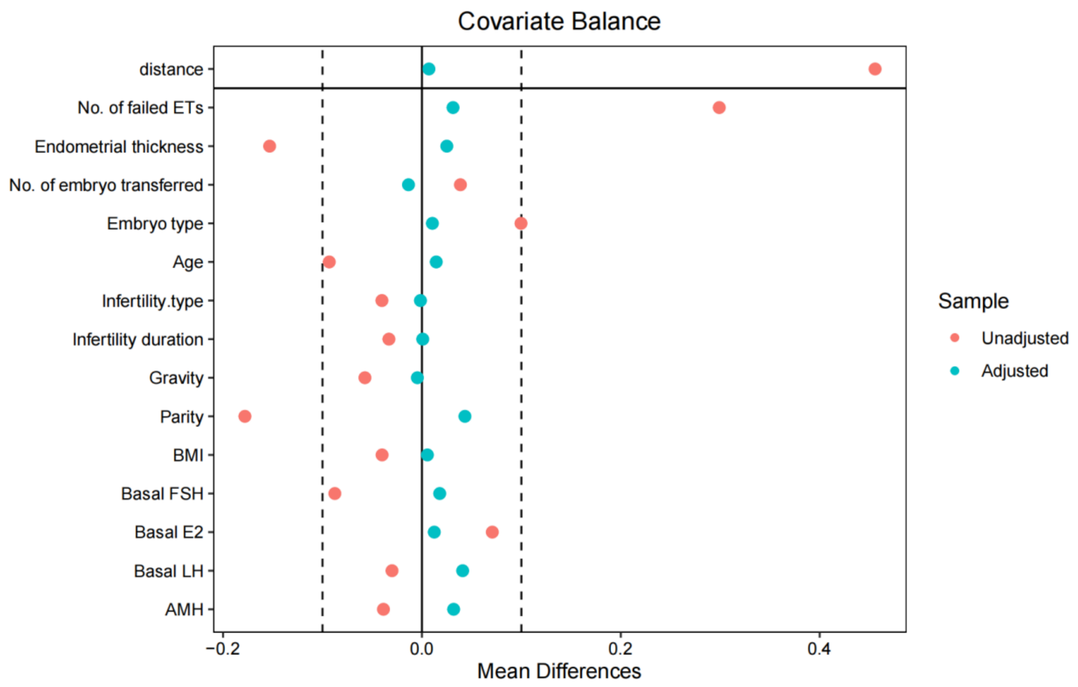

Supplement: Supplementary file 1 [file DataSheet1.pdf]
